# *In silico* structural studies on the vesicular neutral amino acid transporter NTT4 (SLC6A17)

**DOI:** 10.1016/j.csbj.2024.09.004

**Published:** 2024-09-10

**Authors:** Jędrzej Kukułowicz, Marek Bajda

**Affiliations:** Department of Physicochemical Drug Analysis, Faculty of Pharmacy, Jagiellonian University Medical College, Medyczna 9, Krakow 30–688, Poland

**Keywords:** Neutral amino acid transporter NTT4, Glutaminergic regulation, SLC6 family, SLC6A17

## Abstract

NTT4 is one of the neutral amino acid transporters that regulate neural concentration of precursors for glutamate biosynthesis. Here, we provide insight into the structure of NTT4 and rationalize substrate selectivity. Furthermore, we demonstrate how the mutations associated with mental disabilities imply malfunction of the transporter at the molecular level. We also compared the structures of NTT4 and B^0^AT2 (SLC6A15), which is a close homolog, sharing 66 % of the common amino acids. Our analyses may be useful in the search for compounds that inhibit substrate transport. Moreover, they allow a better understanding of the function of these transporters.

## 1 Introduction

The sodium-dependent membrane transporter NTT4 (SLC6A17), a member of the neutral amino acid transporter subfamily (NAATS) from the SLC6 family, is widely distributed within the central nervous system (CNS). This transporter is located primarily in membrane of synaptic vesicles of neurons forming the glutaminergic and GABAergic systems in regions of brain involved in the regulation of learning, memory, mood, and food intake [1], [2]. *In vitro* experiments have shown that NTT4 *via* sodium-driven symport mediates transport of the neutral amino acids, such as leucine, proline, methionine, and glutamine with a transport stoichiometry of 1 amino acid: 1 Na^+^ [3], [4]. However, a recent *in vivo* studies have revealed that the main role of SLC6A17 is to supply synaptic vesicles with glutamine [2]. Furthermore, expression of functional NTT4 is critical for synaptogenesis and neuritogenesis during prenatal development, whilst congenital inherited mutations of NTT4 were found in human individuals manifesting intellectual disability [5]. Intriguingly, it has been shown that virally suppressed SLC6A17 in adult mice resulted in increased levels of γ-aminobutyric acid (GABA) and glutamate in synaptic vesicles [2]. Such a phenomenon may be the result of a shift in the balance of the glutamine-glutamate-GABA metabolism towards the synthesis of neurotransmitters in the cytosol due to the local accumulation of glutamine. Thereby, inhibition of NTT4 might be relevant for synaptic amplification through glutamine-derived neurotransmitters.

In this report, we provide insight into the structure of NTT4 transporters and discuss their structural features that enable transport and discriminate substrate selectivity. In addition, we compare models of NTT4 and B^0^AT2 (SLC6A15), a close homolog with overlapping substrate and profile expression, whose inhibition is suggested to alleviate anxiety and depression [6], [7], [8], [9], [10], [11]. For these purposes, we exploited models of mentioned transporters obtained through homology modeling and derived from AlphaFoldDB (for details on the alignment, model building and assessment process, see methods in the Supporting Information (SI)). In this way, the results of our structural investigations are intended to aid in the rational design of selectively inhibiting compounds. Such compounds will be useful for determining the physiological role and therapeutic relevance of the targeted transporters. It is worth emphasizing here that so far non-substrate inhibitors of NTT4 remain unknown. In addition, due to performed molecular dynamics (MD) simulations, we rationalized the effects of the pathogenic mutations at the molecular level. These findings also contribute to the mechanistic understanding of protein anchoring in the membrane and substrate transport.

## 2 Results and discussion

**Fig. 1:**
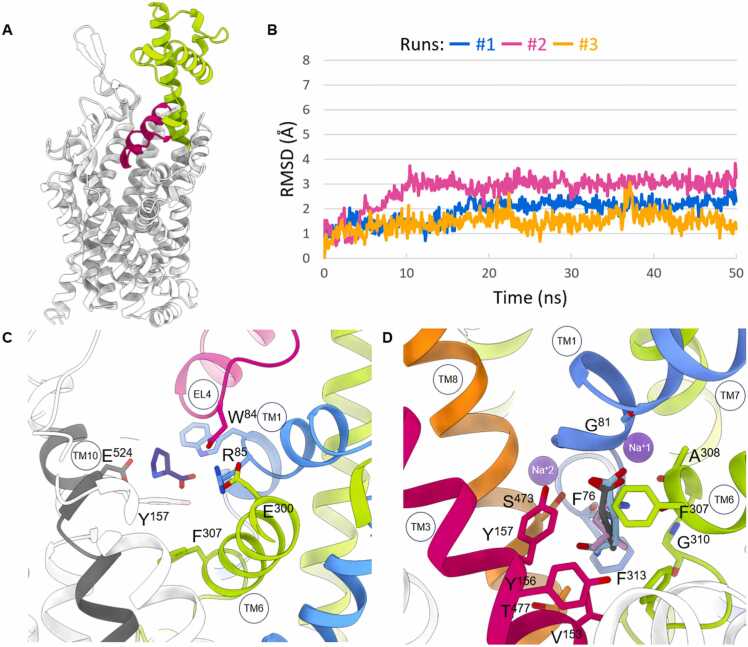
Panel (A) illustrates a model of NTT4 derived from AlphaFoldDB. EL4 is highlighted. The lime green section of EL4 represents a fragment following TM7, while darker pink refers to the V-shaped part. Panel (B) displays RMSD values for proline complexed with NTT4 in an occluded state during three runs of MD simulations. Panel (C) demonstrates proline docked in the S2 site of the outward-open state of the NTT4 transporter. Panel (D) presents the proline (dark gray), leucine (salmon) and glutamine (light blue) docked to the S1 site of NTT4 model in an occluded conformation.

Since AlphaFold has proven to be useful for the recognition of protein folding, we have used the deposited model of NTT4 to study its global architecture [12]. This transporter consists of 12 helical transmembrane domains connected by extracellular (EL) and intracellular (IL) loops and shares the so-called LeuT fold [13]. Moreover, NTT4, like its resolved homologs B^0^AT1 and SIT1 (SLC6A19 and SLC6A20, respectively), stands out by a significantly elongated EL4. Additionally, extended EL3 and EL6 were observed [14], [15], [16], [17]. Interestingly, EL4 of NAATS transporters consists of 2 distinct fragments: one is the elongated helix following TM7, while the other is a V-shaped fragment directed toward the substrate transport pathway (Fig. 1A). It is noteworthy that in the cryo-EM structures of both B^0^AT1 and SIT1, the mentioned helical fragment after TM7 was found to form polar interactions with angiotensin-converting enzyme 2 (ACE2), stabilizing a heteromeric complex. In this context, ACE2 is an auxiliary protein that allows these transporters to be properly expressed in the membrane. Although NTT4 might not interact with ACE2 due to the lack of conserved residues that could form such a heteromeric complex, we hypothesize that well-targeted mutagenesis might allow the generation of an NTT4-ACE2 conjugate that could be helpful for cryo-EM studies, as bulkier proteins are easier to resolve.

In subsequent studies of the determinants that drive the amino acid transport machinery of NTT4, we used structures derived through homology modeling. This approach was dictated by the analysis of AlphaFold models of SLC6 transporters, which show ambiguous conformations compared to resolved SLC6 structures. As a result, these models may lead to false positive interpretations regarding the functionality of amino acid residues that are crucial for the transport mechanism. Although homology models may sometimes fail to provide the correct conformation of the entire modeled protein backbone or residue side chains due to the evolutionary divergence between template and target, they can be more reliable than AlphaFold models. This notion is supported by the fact that sites important for transport tend to be well conserved. In addition, homology models have recently been successfully used for more detailed structural studies [18]. Furthermore, some structural shortcomings of models can be mitigated by combining different computational approaches, *e.g. ab initio* modeling or molecular dynamics (MD) simulations.

According to the transport mechanism, substrate and ions passing through NTT4 interact with the residues distributed along the transport pathway, causing conformational changes. These residues form the vestibule, the extracellular gate, the main substrate binding site (S1), and ion pockets adjacent to the S1 site [19]. The transport of amino acids through NTT4 might begin with the influx of two sodium ions and their accommodation in pockets near the S1 site, creating a favorable electrostatic environment for the subsequent binding of the substrate within the orthosteric site. Since the resolved structures of SLC6 transporters and their bacterial homolog, *Aquifex aeolicus* leucine transporter (aLeuT), present the occluded state while complexed with substrate and co-transported ions, we examined the ion pockets of an NTT4 model in this conformation. This model was built using MODELLER, based on the structures of aLeuT and B^0^AT1 in the occluded conformations as templates (PDB codes: 2a65 and 6m17, respectively) [15], [20]. The resulting model shows that one sodium ion (Na^+^1) is surrounded by TM1, TM6, and TM7, while the other (Na^+^2) interacts with TM1 and TM8. Both ions are coordinated by four groups from the protein. It is worth pointing out that the aLeuT template mentioned above has higher coordination numbers for the corresponding sodium ions. Furthermore, if substrate is bound it provides extra interaction with Na^+^1. Interestingly, mutagenesis studies on GABA transporter 1 (GAT1, SLC6A1) have shown that introducing mutations that affect sodium ion stabilization by reducing the number of contacts results in the loss of substrate transport [21], [22]. By studying other neutral amino acid transporter subfamily members, we have observed that B^0^AT1, like NTT4, shows the same transport stoichiometry [23]. Going forward, we checked the hypothetical coordination number for Na^+^1 and Na^+^2 in B^0^AT1. Surprisingly, insight into the sodium pockets of the B^0^AT1 based on the multiple sequence alignment of the SLC6 family, cryo-EM B^0^AT1 structure in the occluded state (PDB: 6m17) as well as our model built using aLeuT in the occluded state (PDB: 2a65) suggest an ion coordination similar to that of aLeuT. Above observation leads us to speculate that two sodium ions may be harbored in NTT4 pockets near the S1 site, initiating substrate transport turnover. To reinforce this hypothesis, we performed 50 ns MD simulations, repeated three times, using DESMOND after docking of proline to the S1 site of the NTT4 model in its occluded state with two retained sodium ions. Docking was performed in Glide (Schrödinger Suite)(for details on the model building, docking studies and MD simulations, see the Methods section in the SI). The shorter duration of these simulations was intentionally set to track whether ions are kept in a transporter along with the substrate during the MD simulations, rather than to observe substrate and ion translocation. As a result, proline and ions remained stable within their pockets, and the transporter maintained an occluded conformation with a consistent trajectory throughout all runs (Fig. 1B). Furthermore, our steered 10 ns MD simulation for proline complexed with B^0^AT2 in an occluded state, which also displays the same transport stoichiometry as NTT4, showed the movement of the substrate coupled with only one sodium ion (Na^+^1) from the S1 site toward the cytosol (data not shown here). It is also worth noting that the X-ray structures of SLC6 carriers in outward-open or occluded states sometimes contain an ion number inconsistent with the transport stoichiometry (*e.g.* serotonin transporter (SERT), PDB codes: 5i75 and 5i6x) [24].

To discriminate initial substrate recognition, we docked proline to the vestibule of NTT4 using Glide (for details on the docking studies, see methods in the SI). This pocket, recognized as the secondary substrate binding site (S2), has been shown to transiently accommodate substrates and competitive inhibitors [25], [26]. Taking into account that the transporter remains accessible to the substrate in an outward-open state, in this investigation we used the NTT4 model built in the SWISS-MODEL, using the *Drosophila melanogaster* dopamine transporter (dDAT, SLC6A3) in the same conformation as a template (PDB code: 4px9). In the secondary binding site, the amine and carboxyl groups of proline formed ionic and hydrogen bonds with the side chains of Asp524 (TM10) and Trp84 (TM1), respectively. In addition, due to proximity to the proline, we observed that the side chain of Gln436 from V-shaped EL4 and charged Arg85 might create a network of polar interactions with substrate’s carboxylic acid moiety (Fig. 1C). Notably, those vestibular aspartic acid and arginine residues are a polar part of the well conserved extracellular gate of SLC6 members and aLeuT.

*In silico* studies on the molecular basis of transporter substrate selectivity were performed using the same model as for the evaluation of the ion pockets. For the record, the S1 site of this model was built on a template of aLeuT complexed with leucine in an occluded state. Therefore, the size of the S1 site in the NTT4 model should closely reflect the real size of the main substrate binding site in this transporter. Six representative amino acids were picked for selectivity studies. Three of them: proline, leucine, and glutamine are substrates. The first two show high affinity to NTT4 and possess a hydrophobic side chain, but they differ in the amine substitution pattern. The third is an major substrate *in vivo* with a polar side chain. The remaining three amino acids are non-substrates: glutamate and lysine with an oppositely charged side chains, as well as tryptophan with a significantly developed aromatic side chain. After processing of the docking results, we found that the S1 site can be organized into two subsites: polar and hydrophobic. These subsites determine interactions with the substrate or competitive inhibitor. We noticed that amino acid backbone of all studied substrates was coordinated by residues from polar subsite of the S1 site in the same way. The carboxylic acid moiety interacted with Gly81 included in TM1, the side chain of Tyr157 from TM3 and Na^+^1, while the amine group formed a hydrogen bond with Phe307 of TM6, as well as cation-π interactions with aromatic rings of Phe76 (TM1), Phe307, and Phe313 from TM6. These results remain in line with the binding mode for amino acid backbone found in other transporters [20], [21], [27], [28]. Upon further scrutiny, we found that the polar subsite is also lined by Ser473 and Thr477 from TM8 as well as Tyr156 from TM3, whereas the main residues of the hydrophobic subsite are Val153 (TM3), phenylalanines (76, 307, 313) as well as Ala308 (TM6). By studying leucine and proline bound to NTT4, we found that their side chains are surrounded by residues of the hydrophobic subsite. Examination of the NTT4 in complex with glutamine revealed that an amide moiety of substrate directs the side chain toward Thr477 of TM3, but no specific interaction was noticed. These results may explain the higher selectivity of NTT4 towards hydrophobic amino acids over polar ones (Fig. 1D). Non-substrate lysine was docked to our NTT4 model, causing steric clashes within the S1 site, while tryptophan was found beyond this pocket. In the case of glutamate, we found an inconsistent binding mode of the amino acid backbone for five docked poses which might explain no affinity of this amino acid. Interestingly, Thr477 from TM8 of NTT4, in a B^0^AT1 and SIT1 is replaced by Asn (435 and 410, respectively). The presence of Asn435 was shown to stabilize the side chain of glutamine in complex with B^0^AT1, a low affinity primary amino acid transporter. Whereas the Asn410Ala mutant of SIT1 increased the transport of leucine [14], [15]. Notably, SIT1 transporter is specialized to transport glycine and proline. In conclusion, we speculate that Thr477 of NTT4 may implicate the higher substrate promiscuity, however further biochemical validation is required. In the context of proline binding to NTT4, it is worth looking at the unwound fragment of TM6, since Ser280 from this domain of B^0^AT1 seems to exclude this transporter's preference for imino acids [14]. In the determined structures of SIT1 in complex with proline or pipecolate, a close analog of imino acid proline, as well as in our NTT4 model, Gly (253 and 310, respectively) was found in an analogous position [14], [17]. Such a layout may facilitate the transport of proline by NTT4. Among factors that determine size of substrate, it is worth noting that studies on MhsT, a bacterial homolog of SLC6, showed that the unwound fragment of TM6 contains a volumetric sensor revealing the preference for aromatic amino acids [27]. In light of the above, in NTT4 Phe313 of larger size may prevent the accommodation of more bulky amino acid such as tryptophan.

Since the sequence alignment of NTT4 and B^0^AT2 shows 66 % similarity, we examined the B^0^AT2 models to assess structural homology. Upon further analysis, we found that the residues constituting the S2 site, the extracellular gate, and the main substrate binding site of these transporters are identical. Interestingly, research on SLC6A15 inhibitors revealed that loratadine inhibited the uptake of the high-affinity substrate – proline with an IC_50_ of 4 μM. In contrast, the presence of loratadine did not affect substrate transport through NTT4 (IC_50_ > 80 µM) [29]. Due to observed effects, we searched for factors that could explain the aforementioned differences in inhibitory potency between these transporters. Given that SLC6 transporters can be inhibited in all conformations, outward-open, occluded, and inward-open, and having found that B^0^AT2 and NTT4 share the same residues facing the orthosteric site, we hypothesized that loratadine might allosterically inhibit B^0^AT2 and cause the transporter to adopt an inward-open state. Supporting this hypothesis, no kinetic studies of the mode of inhibition have been reported. Loratadine is a relatively large, flat, and rigid compound as the tricyclic aromatic fragment is linked to a hydrophobic six-membered heterocycle with a polar tail by a double-bond. It is a second generation antihistaminergic drug. Despite crossing the blood-brain barrier and being membrane permeant, loratadine does not exhibit psychotropic activity because it is a substrate for P-glycoprotein [30], [31]. As a result, it is possible that loratadine may be a substrate of other membrane transport systems regulating intracellular access. Moreover, allosteric inhibition of B^0^AT2 by the discussed inhibitor may occur in a manner similar to that of tiagabine, which stabilizes the inward-open conformation of GAT1 [32]. Notably, loratadine also inhibited proline uptake through GAT1 with IC_50_ of 10 μM [29]. In searching for differences in the composition of the cytosolic-facing vestibule, we compared the substrate release pathway of NTT4 and B^0^AT2 using models generated on the template of the inward-open GAT1 (PDB code: 7sk2). This site for NTT4 and B^0^AT2 was defined as the residues corresponding to those embracing tiagabine within a 5 Å radius from the mass center of the inhibitor in the GAT1 structure. Astoundingly, residues lining the pathway from the S1 site toward the cytosol in both NTT4 and B^0^AT2 were identical. These results, showing the same layout of the substrate translocation pathway, remain puzzling in the context of factors that limit binding of loratadine to NTT4.

**Fig. 2:**
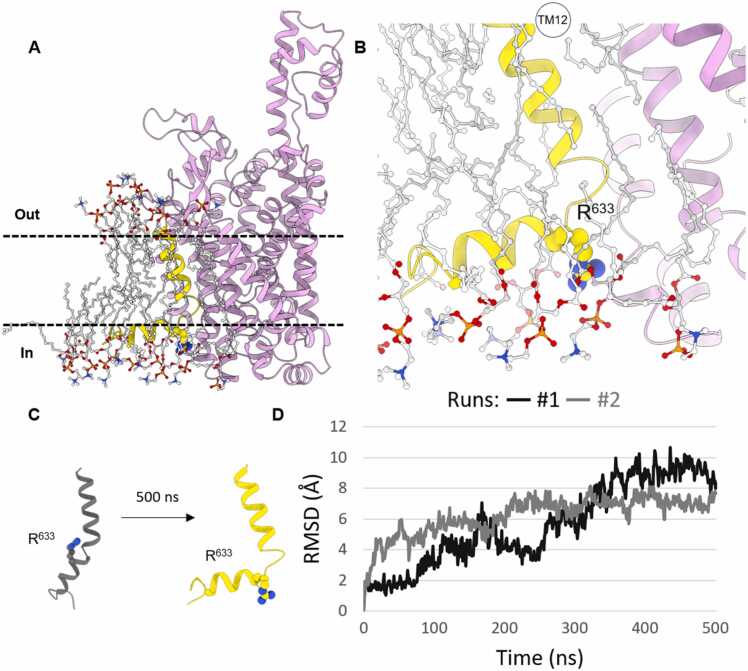
Panels (A) and (B) show a snapshot from a 500 ns MD trajectory of the NTT4 Pro633Arg mutant inserted into a POPC membrane. The transporter is marked in pink and the Pro633Arg mutation is visualized in a yellow marked TM12, which is surrounded by POPC. The interactions of the Arg633 side chain with the polar fragments of the membrane bilayer are shown. Panel (C) demonstrates the conformational change of TM12 between the starting complex of the Pro633Arg NTT4 mutant and after 500 ns of run #1 of the MD simulation. Panel (D) shows the change in the RMSD value for TM12 in runs #1 and #2 of 500 ns MD simulations.

Functional NTT4 is critical for proper brain development during the prenatal stage. Inherited congenital mutations of this transporter cause its aberrant expression or dysfunction, resulting in mental retardation. To investigate molecular determinants of their pathogenicity, we performed a series of MD simulations using DESMOND. The Pro633Arg and Gly162Arg NTT4 mutants were generated in BioLuminate - Residue Scanning and Mutation (Schrödinger Suite). The Pro633Arg mutation was studied based on the NTT4 model derived from AlphaFoldDB (for details on methods, see the SI). Initially, we conducted three 500 ns MD simulations for the WT model as a reference. The choice of such a long simulation time allowed us to distinctly confirm the stability of the transporter in the membrane. In this experiment, the SLC6A17 transporter remained stable in the POPC lipid bilayer in all repetitions and no abnormalities were observed. In further analysis, the charged side chain of arginine in the Pro633Arg mutant, located in the middle of TM12, was shown to be exposed to the hydrophobic tails of the membrane bilayer, suggesting difficulties in anchoring of the transporting protein. To confirm this notion, we performed three replicates of MD simulations for the membrane complex of the mentioned NTT4 mutant, each lasting 500 ns. In all iterations, we noticed a rapid displacement of POPC molecules in the bilayer surrounding Arg633, resulting in interactions of the arginine side chain with polar fragments of phospholipids, thus causing membrane disintegration. Furthermore, in two runs of the MD simulation, we observed significant misfolding of TM12, resulting in an approximately 90° TM kink in its mid-section near the mutation (Fig. 2). Our studies have confirmed at the molecular level the previously reported experimental findings regarding the pathogenicity of this mutation, which revealed the inability of the transporter to anchor to the membrane of the vesicle [2], [5].

**Fig. 3:**
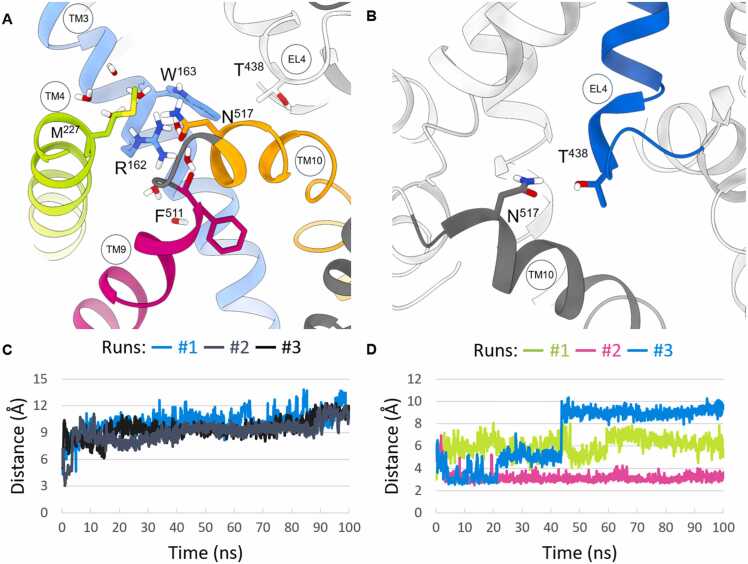
Panels (A) and (B) show a snapshot from a 100 ns MD simulation of NTT4 Gly162Arg mutant (run #1) and WT (run #2). Panel (A) shows polar interactions of residues in the vicinity of the Gly162Arg. Panel (B) illustrates hydrogen bonding, which is thought to stabilize the WT transporter in an inward-open state obtained from the last frame of 100 ns trajectory of run #2. Panel (C) shows the distance between the side chain heavy atoms of Thr438 and Asn517 found in the MD simulations for the Gly162Arg NTT4 mutant, while panel (D) refers to the distance between the heavy atoms of the same residues found in the MD trajectory of the WT NTT4.

The Gly162Arg mutation of SLC6A17 resulted in arginine being positioned in the upper part of TM3 near the V-shaped fragment of EL4, with its side chain exposed on TM4 and TM9. A recent report showed that this NTT4 mutant, despite approaching the membrane, is unable to collect glutamine in the vesicles [2]. Due to the proximity of the mutated residue to EL4, we speculate that the Gly162Arg mutation might interfere with EL4, preventing its native role of stabilizing the transporter in an inward-open conformation. This hypothesis was examined through three 100 ns MD simulations employing a model of the NTT4 mutant in the inward-open state, constructed in MODELLER based on a template of the GAT1 transporter (PDB code: 7sk2). The set simulation time allowed to clearly observe the effects of the mutation without the need to extend it. As a result, we noticed that at the local level in all MD experiments the side chain of Arg162 induced solvation and formed a network of polar interactions with side chains of adjacent Trp163 and Met227 (TM4), main chain of Phe511 included in TM9, and side chain of Asn517 (TM10) (Fig. 3A and Fig. 3C). Our attention was particularly drawn to Asn517 since the side chain of this residue was found to create polar interaction with side chain of Thr438 from EL4 in the initial complex suggesting stabilization of the transporter in inward-open state. Indeed, MD trajectory showed that the presence of charged Arg162 led to the disruption of the specific interaction between Asn517 and Thr438. Thus, the global effect of the mutation would abolish the transport cycle by preventing the transition of the transporter to the inward-open conformation. Aforementioned assumptions were validated by three runs of 100 ns MD simulations for WT NTT4 using the same experimental conditions as for the mutant. The validation process confirmed the involvement of Asn517 in maintaining the transporter in an inward-open state by the interaction with Thr438 (Fig. 3B and Fig. 3D).

It is worth emphasizing that recently published cryo-EM structures of SLC6 transporters remain in an occluded or inward-open state, plausibly due to experimental conditions. Thus, our observations suggest that targeted mutagenesis of other SLC6 carriers at the corresponding position of Asn517 or Gly162 of NTT4 might be useful in obtaining the transporter in an outward-open state, assuming that these residues play a role in stabilizing the mentioned conformations.

## 3 Conclusions

In summary, our structural studies shed light on the molecular determinants of substrate selectivity for NTT4. Primarily, hydrophobic component of main substrate binding site (S1) of SLC6A17 discriminates substrate selectivity as it is capable of accommodating neutral amino acids. Furthermore, our models showed that residues lining the substrate transport pathway of NTT4 and B^0^AT2 are the same. This, likely accounts for their similar substrate profile, as observed in *in vitro* studies. However, unidentified molecular factors contribute to the selectivity of loratadine against B^0^AT2 over NTT4. Furthermore, our MD simulation studies on pathogenic mutations of NTT4 suggest that Pro633Arg leads to misfolding of TM12, which impairs membrane anchoring, whereas Gly162Arg may be associated with insufficient EL4 movements, which are essential for the alternating-access transport mechanism.

## 4 Methods

The methods: sequence alignment, model sourcing and model evaluation, docking studies and molecular dynamics simulations are described in the Supporting Information.

## Funding sources

This research was supported by the Strategic Program Excellence Initiative at Jagiellonian University, Research Support Module (U1C/W42/NO/28.19), Visibility and Mobility Module, and Jagiellonian University Medical College Grant (N42/DBS/000375).

## Declaration of competing interests

There are no conflicts to declare.

## CRediT authorship contribution statement

**Marek Bajda:** Writing – review & editing, Supervision, Funding acquisition, Formal analysis, Conceptualization. **Jędrzej Kukułowicz:** Writing – review & editing, Writing – original draft, Investigation, Funding acquisition, Conceptualization.

## Declaration of Competing Interest

The authors declare that they have no known competing financial interests or personal relationships that could have appeared to influence the work reported in this paper.
