# Supplementary material for: In silico structural studies on the vesicular neutral amino acid transporter NTT4 (SLC6A17)
Source: Comput Struct Biotechnol J. 2024 Sep 10;23:3342–7. doi: 10.1016/j.csbj.2024.09.004 (PMC11416165; doi:10.1016/j.csbj.2024.09.004)
Supplement: Supplementary file 1 — Supplementary material. [file mmc1.docx]

**Supporting Information**

*In silico* structural studies on the vesicular neutral amino acid transporter NTT4 (SLC6A17)

Jędrzej Kukułowicz ^1^ and Marek Bajda ^1,*^

^1^ Department of Physicochemical Drug Analysis, Faculty of Pharmacy, Jagiellonian University Medical College, Cracow, Poland

^*^Correspondence to: [marek.bajda@uj.edu.pl](mailto:marek.bajda@uj.edu.pl)

**Table of Contents**

[**1.** **Methods** 1](#_Toc164888664)

[**1.1.** **Sequence alignment, model sourcing and model evaluation** 1](#_Toc164888665)

[**1.2.** **Docking studies** 2](#_Toc164888666)

[**1.3.** **Molecular dynamics simulations** 2](#_Toc164888667)

[**2.** **References** 3](#_Toc164888668)

# **Methods**

## **Sequence alignment, model sourcing and model evaluation**

The NTT4 and B^0^AT2 sequence was obtained from the Uniprot Database (entry codes: Q9H1V8 and Q9H2J7 respectively) ^1^. Initially, ClustalOmega was used to perform multiple sequence alignments of SLC6 transporters and aLeuT ^2^. Then, pairwise alignments were compared with the results of the multiple sequence alignment and with findings from other reports ^3^. Adjustments were made to the alignment of the investigated transporters and aLeuT as needed.

The unmodified crystal structure of dDAT in the outward-open state (PDB code: 4xp9) and the cryo-EM structure of GAT1 in the inward-open state (PDB code: 7sk2) were used as templates to construct NTT4 and B^0^AT2 models in the corresponding conformations. To build models of studied transporters in the occluded state, a chimeric aLeuT-B^0^AT1 template was constructed using the structure of the aLeuT transporter in the occluded state (PDB: 2a65) as the core and the fragment of EL4 of the B^0^AT1 transporter in the occluded state obtained by cryo-EM method (PDB: 6m17). To this end, B^0^AT1 has been superimposed with aLeuT, then the helical fragment of the EL4 following TM7 was extracted from B^0^AT1 (PDB code: 6m17, chain A) and transferred to the aLeuT. Therefore, in the chimeric aLeuT template, original V-shaped conformation of EL4 directed towards the entrance cavity was retained. In addition, the proposed chimeric design is unlikely to influence ligand binding, as the transferred fragment of EL4 does not contribute to substrate interactions with the transporter.

Using the AutoModel class in MODELLER 10.1, 50 models of the investigated transporters in occluded and inward-open conformations were generated using appropriate of the mentioned templates ^4^. Also, the sequence alignment input file was missing the N- and C-termini, where the corresponding amino acids of the templates are missing. Subsequently, 10% of the models with the highest DOPEscore were selected for further evaluation using QMEAN, Ramachandran plot, and Verify3D ^5,6^.

SWISS-MODEL was used to generate models of the investigated transporters in the outward-open state ^7^. These models were evaluated in the same way as the models created in MODELLER.

The NTT4 and B^0^AT2 AlphaFold models were derived from AlphaFoldDB (entry codes: Q9H1V8 and Q9H2J7, respectively) ^8^.

Visualization of the models was performed with PyMOL, Maestro and UCSF ChimeraX ^9^.

## **Docking studies**

Docking studies were carried out using the Schrödinger Suite release 2020-3. Studied amino acids were prepared with the LigPrep module with default options. Ionization was predicted for the pH value of 7.4 ± 0.2. The models were prepared with the Protein Preparation Wizard with default options.

Docking of appropriate amino acids was performed to the NTT4 model in an occluded and outward-open state. In the case of docking to the NTT4 model in an occluded state, the center of the grid was defined by the center of mass of Gly81, Val153, and Ser477, which correspond to residues lining the main substrate binding site in aLeuT (Gly24, Val104, and Ser355). The size of the grid box for amino acid docking to the transporter in the occluded state was 15Å. In the case of docking to the NTT4 model in an outward-open state, the center of the grid was designated as the mass center of Thr438, Asp524, and Arg85. These residues correspond to the amino acids facing the vestibule found in aLeuT (Phe320, Asp404 and Arg30). The size of the grid box for L-proline docking to the transporter in the outward-open state was 10Å.

All docking studies were performed with standard precision. Five poses were stored for each ligand and post-docking minimization was performed. The OPLS3e force field was used for all of the above calculations.

## **Molecular dynamics simulations**

All of the MD simulation experiments were carried out using the following protocol. The complexes subjected to MD experiments were positioned within the membrane using the OPM server ^10^. MD systems for simulations were constructed using the System Builder module within the Schrödinger Suite 2020-3, utilizing TIP3P water and POPC membrane models. The protein-ligand complex was positioned within an orthorhombic box measuring 15Å×15Å×15 Å. To neutralize the system, Na^+^ and Cl^-^ ions were added to achieve a final concentration of 0.15 M. The system underwent minimization and pre-equilibration using the standard protocol in DESMOND 6.3. A final production simulation of the ascertained duration was then performed in the NPT ensemble at 300 K and 1.013 bar, using a time step of 2 fs and a recording interval of 100 ps. The seed was set to random, while other parameters were left at their default values. The simulation was performed using the OPLS3e force field.

# **References**

1 A. Bateman, M. J. Martin, S. Orchard, M. Magrane, R. Agivetova, S. Ahmad, E. Alpi, E. H. Bowler-Barnett, R. Britto, B. Bursteinas, H. Bye-A-Jee, R. Coetzee, A. Cukura, A. da Silva, P. Denny, T. Dogan, T. G. Ebenezer, J. Fan, L. G. Castro, P. Garmiri, G. Georghiou, L. Gonzales, E. Hatton-Ellis, A. Hussein, A. Ignatchenko, G. Insana, R. Ishtiaq, P. Jokinen, V. Joshi, D. Jyothi, A. Lock, R. Lopez, A. Luciani, J. Luo, Y. Lussi, A. MacDougall, F. Madeira, M. Mahmoudy, M. Menchi, A. Mishra, K. Moulang, A. Nightingale, C. S. Oliveira, S. Pundir, G. Qi, S. Raj, D. Rice, M. R. Lopez, R. Saidi, J. Sampson, T. Sawford, E. Speretta, E. Turner, N. Tyagi, P. Vasudev, V. Volynkin, K. Warner, X. Watkins, R. Zaru, H. Zellner, A. Bridge, S. Poux, N. Redaschi, L. Aimo, G. Argoud-Puy, A. Auchincloss, K. Axelsen, P. Bansal, D. Baratin, M. C. Blatter, J. Bolleman, E. Boutet, L. Breuza, C. Casals-Casas, E. de Castro, K. C. Echioukh, E. Coudert, B. Cuche, M. Doche, D. Dornevil, A. Estreicher, M. L. Famiglietti, M. Feuermann, E. Gasteiger, S. Gehant, V. Gerritsen, A. Gos, N. Gruaz-Gumowski, U. Hinz, C. Hulo, N. Hyka-Nouspikel, F. Jungo, G. Keller, A. Kerhornou, V. Lara, P. Le Mercier, D. Lieberherr, T. Lombardot, X. Martin, P. Masson, A. Morgat, T. B. Neto, S. Paesano, I. Pedruzzi, S. Pilbout, L. Pourcel, M. Pozzato, M. Pruess, C. Rivoire, C. Sigrist, K. Sonesson, A. Stutz, S. Sundaram, M. Tognolli, L. Verbregue, C. H. Wu, C. N. Arighi, L. Arminski, C. Chen, Y. Chen, J. S. Garavelli, H. Huang, K. Laiho, P. McGarvey, D. A. Natale, K. Ross, C. R. Vinayaka, Q. Wang, Y. Wang, L. S. Yeh, J. Zhang, P. Ruch and D. Teodoro, *Nucleic Acids Res*, 2021, **49**, D480–D489.

2 F. Sievers, A. Wilm, D. Dineen, T. J. Gibson, K. Karplus, W. Li, R. Lopez, H. McWilliam, M. Remmert, J. Söding, J. D. Thompson and D. G. Higgins, *Mol Syst Biol*, 2011, **7**, 539.

3 T. Beuming, L. Shi, J. A. Javitch and H. Weinstein, *Mol Pharmacol*, 2006, **70**, 1630–1642.

4 B. Webb and A. Sali, *Curr Protoc Bioinformatics*, 2016, **2016**, 5.6.1-5.6.37.

5 G. Studer, C. Rempfer, A. M. Waterhouse, R. Gumienny, J. Haas and T. Schwede, *Bioinformatics*, 2020, **36**, 1765–1771.

6 D. Eisenberg, R. Lothy and J. U. Bowie, *Methods Enzymol*, 1997, **277**, 396-404.

7 A. Waterhouse, M. Bertoni, S. Bienert, G. Studer, G. Tauriello, R. Gumienny, F. T. Heer, T. A. P. De Beer, C. Rempfer, L. Bordoli, R. Lepore and T. Schwede, *Nucleic Acids Res*, 2018, **46**, W296–W303.

8 J. Jumper, R. Evans, A. Pritzel, T. Green, M. Figurnov, O. Ronneberger, K. Tunyasuvunakool, R. Bates, A. Žídek, A. Potapenko, A. Bridgland, C. Meyer, S. A. A. Kohl, A. J. Ballard, A. Cowie, B. Romera-Paredes, S. Nikolov, R. Jain, J. Adler, T. Back, S. Petersen, D. Reiman, E. Clancy, M. Zielinski, M. Steinegger, M. Pacholska, T. Berghammer, S. Bodenstein, D. Silver, O. Vinyals, A. W. Senior, K. Kavukcuoglu, P. Kohli and D. Hassabis, *Nature*, 2021, **596**, 583–589.

9 T. D. Goddard, C. C. Huang, E. C. Meng, E. F. Pettersen, G. S. Couch, J. H. Morris and T. E. Ferrin, *Protein Sci*, 2018, **27**, 14–25.

10 M. A. Lomize, I. D. Pogozheva, H. Joo, H. I. Mosberg and A. L. Lomize, *Nucleic Acids Res*, 2012, **40**, D370-6.
